# Health professionals’ readiness to implement electronic medical record system in Gamo zone public hospitals, southern Ethiopia: an institution based cross-sectional study

**DOI:** 10.1186/s12913-023-09745-5

**Published:** 2023-07-19

**Authors:** Samuel Hailegebreal, Temesgen Dileba, Yosef Haile, Sintayehu Abebe

**Affiliations:** 1 College of Medicine and Health Sciences, School of Public Health, Department of Health Informatics, Wachemo University, Hosaena, Ethiopia; 2 School of Public Health, College of Medicine and Health Sciences, Arba Minch University, Arba Minch, Ethiopia

**Keywords:** Electronic health record, Electronic medical record, Readiness, Health professional, Southern Ethiopia

## Abstract

**Background:**

The adoption of Electronic Medical Records (EMR) by the healthcare sector can improve patient care and safety, facilitate structured research, and effectively plan, monitor, and assess disease. EMR adoptions in low-income countries like Ethiopia were delayed and failing more frequently, despite their critical necessity. The most popular way to solve the issue is to evaluate user preparedness prior to the adoption of EMR. However, little is known regarding the EMR readiness of healthcare professionals in this study setting. Therefore, the objective of this study was to assess the readiness and factors associated with health professional readiness toward EMR in Gamo Zone, Ethiopia.

**Methods:**

An institution-based cross-sectional survey was conducted by using a pretested self-administered questionnaire on 416 study participants at public hospital hospitals in southern Ethiopia. STAT version 14 software was used to conduct the analysis after the data was entered using Epi-data version 3.2. A binary logistic regression model was fitted to identify factors associated with readiness. Finally, the results were interpreted using an adjusted odds ratio (AOR) with a 95% confidence interval (CI) and p-value less than 0.05.

**Results:**

A total of 400 participants enrolled in the study, with a response rate of 97.1%. A total of 65.25% (n = 261) [95% CI: 0.60, 0.69] participants had overall readiness, 68.75% (n = 275) [95% CI: 0.64, 0.73] had engagement readiness, and (69.75%) (n = 279) [95% CI: 0.65, 0.74] had core EMR readiness. Computer skills (AOR: 3.06; 95% CI: 1.49–6.29), EMR training (AOR: 2.00; 95% CI: 1.06–3.67), good EMR knowledge (AOR: 2.021; 95% CI: 1.19–3.39), and favorable attitude (AOR: 3.00; 95% CI: 1.76–4.97) were factors significantly associated with EMR readiness.

**Conclusion:**

Although it was deemed insufficient, more than half of the respondents indicated a satisfactory level of overall readiness for the adoption of EMR. Moreover, having computer skills, having EMR training, good EMR knowledge, and favorable EMR attitude were all significantly and positively related to EMR readiness.

## Introduction

E-health is defined by the World Health Organization (WHO) as the cost-effective application of information and communication technologies (ICT) to assist health and health-related disciplines [1, 2]. The primary issues facing healthcare systems can be greatly addressed by EMR. The delivery of healthcare services to patients is supported by the use of an electronic medical record (EMR), which is a computerized medical record used to collect, store, and share data among healthcare professionals in an organization.

Although EMRs are a vital tool for the health sector, their implementation, uptake, and utilization are still low in developing nations. Many healthcare facilities throughout the world have deployed EMR systems to enhance the process of capturing patient data, but only a select fraction of them have proven successful [3, 4]. EMRs are computerized medical information systems that gather, store, and display patient data. While maintaining the patient’s privacy and security, it may include a variety of data such as socio-demographics, insurance, past and present medication information, allergies, laboratory and test results, histories of immunizations and medical procedures, hospitalizations, progress evaluations, and others [5–7].

E-Health Readiness is the term used to describe how ready healthcare organizations or communities are for the anticipated change brought about by ICT-related activities. E-readiness is the capacity of an organization to foster and support the development of ICTs, including infrastructure, pertinent systems, and technical competencies [8]. EMR enhances patient care by establishing connections among all caregivers, lowering the demand for file space and supplies, and eliminating the need for staff to physically access any records [9]. However, in many developing countries the EMR system is not widely scattered or implemented [10–13].

Failure to implement EMRs has a negative impact on patients’ and healthcare professionals’ ability to access medical history, treatment information, and past diagnoses, which slows down the workflow of healthcare organizations [14]. The development of a national electronic health record is now underway in Ethiopia, while other EMR pilot programmes have been implemented in our country [15, 16]. Previous finding revealed that the proportion of EMR readiness varied across Ethiopia, with 36.5% [17] in the Sidama region, 52.8% [18] in the southwest Ethiopia, and 54.1% [2] in the northern Ethiopia.

Prior research suggested that readiness was related to age, gender, profession, level of education, and work experience [15, 19–22]. Moreover, having knowledge, computer skills, prior EMR experience, and a personal computer are associated to professional readiness [13, 23–27]. Previous research revealed that readiness for the adoption of EMR can be influenced by health professionals working in organizations with IT infrastructure and access to computers [15, 28]. Our literature search revealed that more studies have focused on organizational readiness than on the health professionals’ readiness [29], and no research has been done in the setting of our study.

Before implementing such systems in Ethiopia, the researchers of this study felt that it was important to assess the user’s readiness and the essential metrics. In environments with limited resources, this study also made it possible for policymakers to comprehend user needs prior to system deployment. Therefore, the objective this study was to assess readiness of health professionals towards electronic medical record system and its associated factors in public hospitals in Gamo zone, Ethiopia.

## Method

### Study design, setting, and period

From September to October 2022, a cross-sectional institution-based survey was undertaken among healthcare professionals working at public health institutions in the Gamo zone. Gamo zone shares a border with Wolayta and Gofa zones in the north, Lake Abaya to the northeast, Amaro and Dirashe special woredas to the southeast, and South Omo to the southwest. The administrative center of the Gamo zone is Arba Minch town. Arba Minch settlement lays 505 km (km) southwest of Addis Ababa, the Ethiopian capital, and 275 km (km) southwest of Hawassa, the regional headquarters of southern Ethiopia. It hosted seven hospitals (one general and six primary hospitals), 56 health centers, and 299 health posts, which serve the community by providing preventive and curative services [30].

### Study population, sample size and sampling procedure

**Fig. 1:**
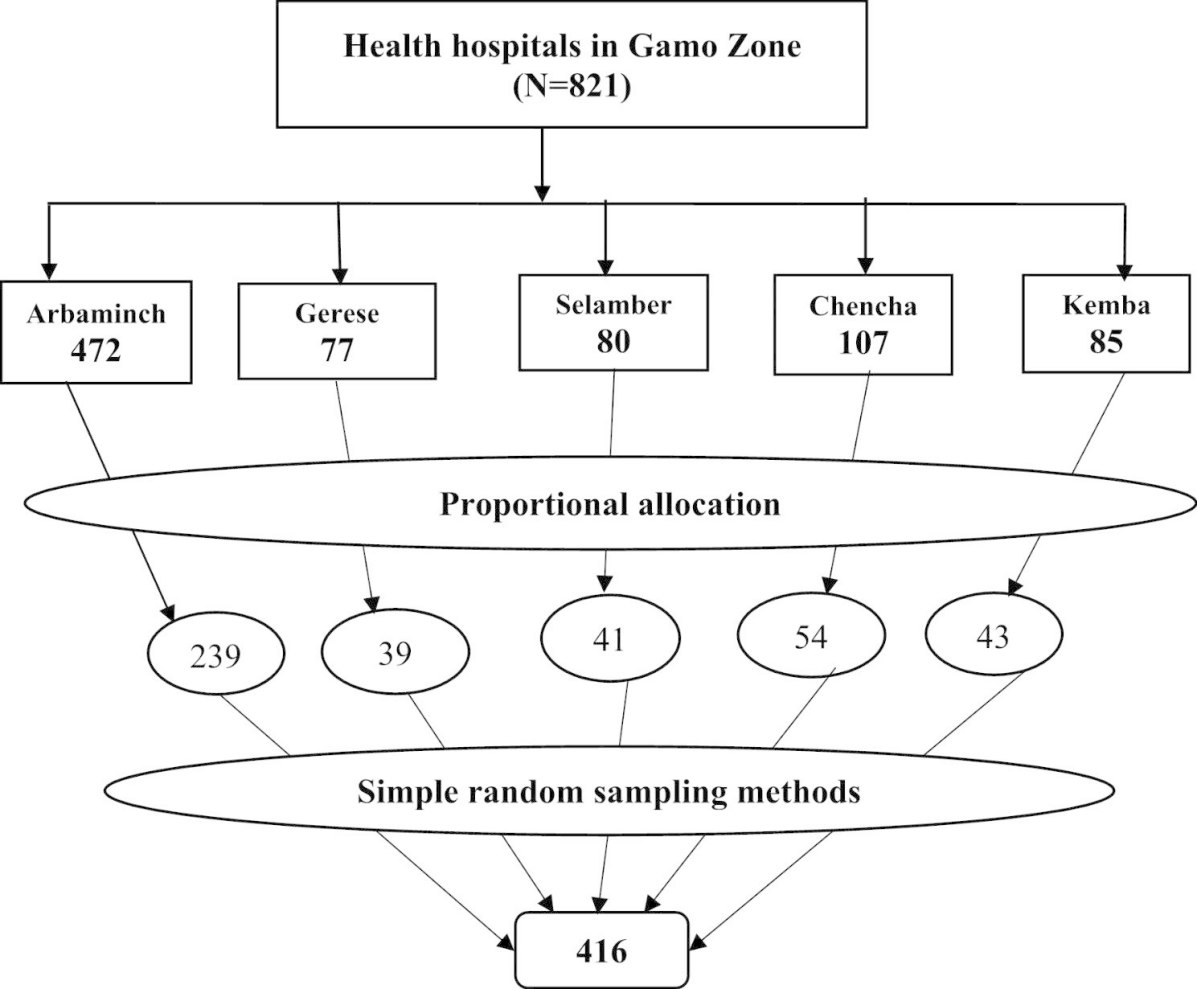
Sampling procedure on EMR readiness among health professionals in Gamo zone, Ethiopia, 2022

All healthcare workers who were employed full-time in the Gamo zones of southern Ethiopia were eligible for this study. Health professionals working in Gamo zone hospitals (Arba Minch, Geress, Selamber, Chencha, and Kemba) were study population. The sample size was calculated using data from a study carried out in Ethiopia, which showed that 52.8% of medical professionals had EHR readiness levels comparable to those used in the current study [18]. We also take into account the following assumptions: a non-response rate of 10%, a margin of error of 5%, and a confidence level of 95%. Finally, the study’s sample size was 416 health professionals. We were proportionally allocated the total sample size, 416, to those five public hospitals found in the zones. Then, in those five hospitals, random selections of healthcare professionals were executed (Fig. 1).

### Data collection technique and data quality assurance

A pretested self-administered questionnaire was used to collect the data. The questionnaire had questions about socio-demographics, behavior, technology, and organizations. The survey was written in English because the study’s participants are well-educated and capable of understanding it. Cronbach’s alpha coefficient was also used to test reliability (the overall Cronbach alpha for healthcare professionals readiness was 0.87, knowledge was 0.81 and attitude was 0.89). For two days, the investigators trained the supervisors and data collectors on the purpose of the study, data gathering techniques, data collection tools, respondents’ approach, data confidentiality, and respondent’s rights. Five health information technicians with strong communication skills were used for data collection, and two master’s-educated health professionals served as supervisors. The supervisors checked the questionnaire’s accuracy every day. Before the analysis, data cleaning and cross-checking were also performed. Following any necessary questionnaire adjustments, the actual data collection process began.

### Operational definition

#### Core readiness

Core readiness was assessed in this study using a series of four questions, with participants scoring 50% or higher being assumed to have core readiness and participants scoring less than 50% being believed to not have core readiness [18].

#### Engagement readiness

Engagement readiness is the active willingness and participation of individuals in the deployment of the electronic medical record (EMR). In this study, participants’ levels of engagement readiness was assessed by a series of four questions. Participants with scores of 50% or more were considered to be engaged, while those with scores averaging less than 50% were considered not to be so [31].

#### Overall readiness

Health professionals were classified as having an overall readiness if they met both the core and engagement readiness criteria [24].

#### Knowledge

Eight questions were used to examine if a person possesses the fundamental understanding of EMR, and knowledge is measured as a variable. Professionals with knowledge scores of 50% or higher were considered to have good knowledge [23].

#### Attitude

Attitude was measured as a latent variable of a set of fifteen questions that assesses the individual perception of EMR measured on a five point Likert scale. A score of mean or above was used to classify as having a favorable attitude [32].

### Data processing and analysis

Data from the survey were entered using Epi-data version 3.1, coded by using alpha-numeric symbols, and analyzed using STATA version 14 software. To describe demographic characteristics, attitudes, and readiness for EMR, descriptive analyses were conducted. Moreover, the binary logistic regression method was used to identify the independent factors related to readiness. The variance inflation factor was used to test for multicollinearity (VIF). Hosmer-Lemeshow tests were used to assess the model’s goodness of fit at P-value > 0.05. In multi-variable logistic regression analysis, odds ratio was used to examine the strength of association between factor and outcome variables and 95% CI and P-value < 0.05 were computed to assess statistical significance.

## Result

### Socio demographic characteristics of study participant

Of the total 416 participants, 400 returned the questionnaire with a response rate of 97.1%.In this study, majority of the participants (53.3%) were within the age category 20–30 and 62% of the participants were male professionals. About 131 (28.25%) respondents were nurses, 72(18%) were doctors, 66(16.50%) were public health, 51(12.75%) were midwives and 69(17.25%) were other heath professional. Of the respondents, 322 (80.50%) have a bachelor degree while 36(9%) health professionals had a Master’s degree and above. About 184 (46%) of the study participants had more than five years of professional experience, while 29 (7.25%) had less than two years. Moreover, 288(72%) have worked at the hospital where they are currently employed (Table 1).

**Table 1:** Socio-demographic characteristics of health professionals working in, Gamo zone, Ethiopia 2022

Variables	Frequency	Percent (%)
**Age**		
20–30	187	46.75
31–40	153	38.25
>=41	60	15.00
**Sex**		
Male	248	62.00
Female	152	38.00
**Educational status**		
Diploma	42	10.50
Degree	322	80.50
MSC and above	36	9.00
**Profession**		
Medicine	72	18.00
Public health	66	16.50
Midwifery	51	12.75
Nurse	113	28.25
Pharmacy	29	7.25
others	69	17.25
**Work experience**		
Less than 2 years	29	7.25
2–3 years	64	15.75
4-5 years	124	31.00
> 5 years	184	46.00
**Duration in current hospital**		
<=12 month	48	12.00
13–24 month	64	16.00
>=24 month	288	72.00
**Salary**		
< 146.6$	167	44.30
= >146.6$	210	57.70

**Key: 1$= 54.40 ETB**

### Organizational and technical factors

Majority (65%) of respondents have a personal computer at home and 337 (84.25%) of the participants had computer related skills. Only 109 (27.25%) of the study’s participants had prior EMR training, and only 616 (41%) had prior EMR system experience. More than half of (52%) the health professionals had access to a computer in their workplace; of these, 63.37% participant used for data recording, 20.30% for report writing, 10.89% for reading, and 5.45% for other purposes like video accessing. Moreover, more than half of the respondents reported that their hospitals have an IT technician and have a functioning IT department (Table 2).

**Table 2:** Technical and organizational factors for health professional readiness in Gamo Zone, Ethiopia 2022

Variable	Frequency	Percent (%)
**Personal computer at home**		
Yes	260	65.00
No	140	35.00
**Computer skills**		
Yes	337	84.25
No	63	15.75
**Previous EMR experience**		
Yes	164	41.00
No	236	59.00
**Functional IT department**		
Yes	218	54.50
No	182	45.50
**IT technical person**		
Yes	258	64.50
No	142	35.50
**Computer access in office**		
Yes	208	52.00
No	192	48.00
**Purpose of computer(n = 208)**		
Data recoding	128	62.37
Report generating	41	20.30
Reading	22	10.89
Video and other	11	5.45

### Knowledge and attitude of health professionals for EMR

**Fig. 2:**
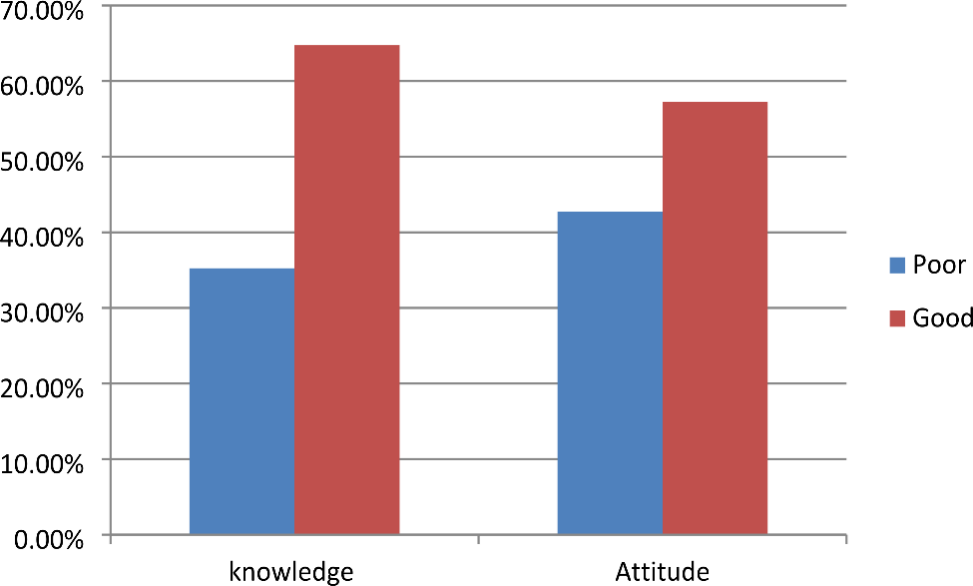
EMR knowledge and attitude towards EMR among health professionals in Gamo zone, Ethiopia, 2022

In this study the majority of respondents 259 (64.75%) had good knowledge of EMR. Likewise, 229 (57.25%) respondents had a favorable opinion of the EMR system (Fig. 2).

### Readiness of health professional to EMR

**Fig. 3:**
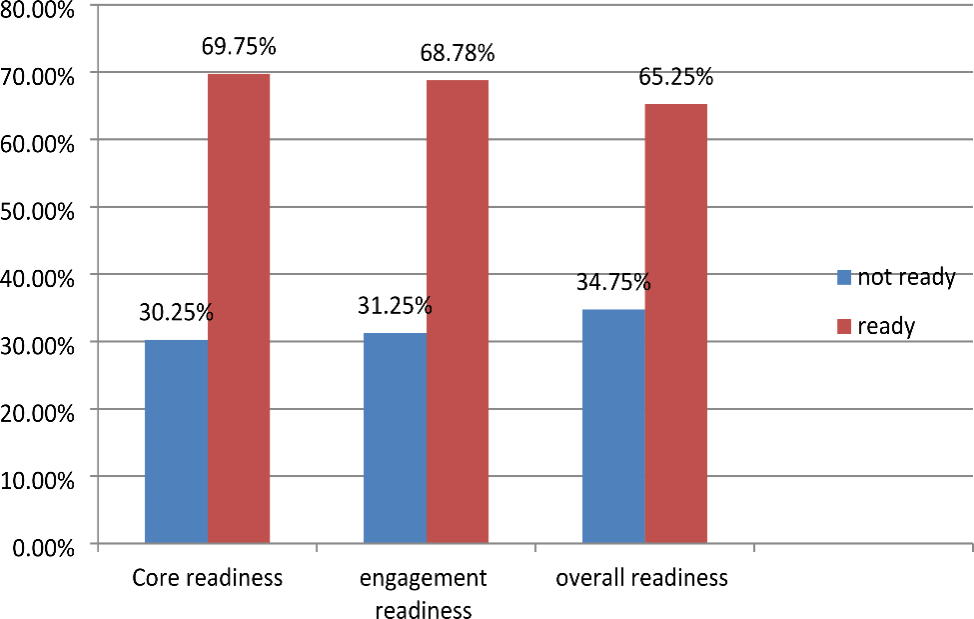
Health professional readiness to implement EMR in public hospitals in Gamo zone, Ethiopia, 2022

Out of all participants, 65.25% [95% CI: 0.60, 0.69] had overall readiness for EMR, while 68.75% [95% CI: 0.64, 0.73] had engagement readiness for EMR and 69.75% [95% CI: 0.65, 0.74] had core readiness (Fig. 3).

### Factors associated with health professional readiness to EMR

In this study, four factors were shown to be significantly associated with EMR readiness in the multivariable logistic regression model: computer skills, EMR training, EMR knowledge, and EMR attitude.

Health care professionals with computer skills were 3 times (AOR: 3.06; 95% CI: 1.49–6.29) more likely to be ready to use an EMR system than their counterparts. Health professionals who had taken EMR training were about 2 times (AOR: 2.00; 95% CI: 1.06–3.67) more likely ready to use EMR system than those who had not. Health professionals with good EMR knowledge had 2times (AOR: 2.021; 95% CI: 1.19–3.39) higher odds of being ready than those with poor knowledge. Moreover, it was revealed that professionals with a favorable attitude had 3 times (AOR: 3.00; 95% CI: 1.76–4.97) more likely ready than those with unfavorable attitude (Table 3).

**Table 3:** Multivariable analysis of factors associated with health professional readiness to EMR in Gamo Zone, Ethiopia 2022

Variables	Overall readiness		COR (95% CI)	AOR (95% CI)
	Ready (%)	Not ready (%)		
**Educational status**				
Diploma	24(57.1)	18(42.9)	1	1
Degree	211(65.5)	111(34.5)	1.43 [0.74,2.73]	0.73[0.311.71]
MSC and above	26(72.2)	10(27.8)	1.95 [0.75,5.04]	0.66[0.19,2.25]
**Profession**				
Medicine	54(75)	18(25)	1	1
Public health	32(48.5)	34(51.5)	0.33[0.15,0 0.64]	0.46[0.19,1.10]
Midwife	33(64.7)	18(35.3)	0.61[0.27,1.33]	1.24[0.49,3.22]
Nurse	67(59.3)	46(40.7)	0.48[0.25, 0.93]	0.96[0.40,2.06]
Pharmacy	23(79.3)	6(20.7)	1.27[0.44,3.63]	1.52[0.47,4.86]
Others	52(75.4)	17(24.6)	1.01[0.47, 2.18]	1.76[0.70,4.41]
**Work experience**				
Less than 2 years	24(82.8)	5(17.2)	1	1
2–3 years	46(73.1)	17(26.9)	0.56[0.18,1.71]	1.15[0.33,3.93]
4–5 years	75(60.5)	49(39.5)	0.33[0.11,0.89]	0.59[0.17,2.06]
> 5 years	116(63.1)	68(36.9)	0.35[0.12,0.97]	0.76[0.20,2.79]
**Duration of service in current institution**				
<=12 month	33(68.7)	15(31.3)	1	1
13–24 month	47(73.4)	17(26.6)	1.25[0.55,2.86]	0.89[0.34,2.29]
>=24 month	181(62.8)	107(37.2)	0.76[0.39,1.48]	0.94[0.39,2.25]
**Computer skills**				
No	22(34.9)	41(65.1)	1	1
Yes	239(70.9)	98(29.1)	4.54[2.57,8.02]*	3.06[1.49, 6.29]**
**Personal computer**				
No	77(55)	63(45)	1	1
Yes	184(70.8)	76(29.2)	[1.29,3.03]**	1.06[0.57,1.95]
**IT technical person**				
No	81(57.1)	61(42.9)	1	1
Yes	180(69.8)	78(30.2)	1.73[1.13,2.65]*	0.91[0.52,1.60]
**EMR training**				
No	184(63.2)	107(36.8)	1	1
Yes	77(70.6)	32(29.4)	1.39[0.86, 2.25]	1.98[1.06,3.67]*
**EMR knowledge**				
Poor	66(46.8)	75(53.2)	1	1
Good	195(75.3)	64(21.7)	3.46[2.24,5.34]**	2.01[1.19, 3.39]**
**Attitude towards EMR**				
Unfavorable	84(49.1)	87(50.9)	1	1
Favorable	177(77.3)	52(22.7)	3.52[2.29,5.42]**	2.95[1.76,4.97]**

*Key: 1: reference group; []: confidence interval, p-value 0.05–0.01 *: p-value < 0.01 **

## Discussion

This study examined the EMR readiness of healthcare professionals and related variables in Southern Ethiopia. Despite the fact that there are several levels of EMR readiness, the authors of this study emphasized on healthcare providers’ levels of readiness. In this study, the overall readiness of health professionals for an EMR system was 65.25%.This finding was similar with study done in Ethiopia (62.3%) [23]. This finding was higher than study conducted in Southwest Ethiopia (52.8%) [18], Sidama region (36.5%) [32], Ghana (54.9%) [24], Myanmar (54.2%) [33]. The discrepancy may be attributable to the development and extension of Technological infrastructure, as well as the Ethiopian government’s priority placement of the digitization of the health information system in its ambition to modernize the health sector [34, 35]. The regular interaction of health workers with the global digital world may also be a factor. Moreover, the method used to categorize professional readiness, variations in sample size, or variations in socio-demographic factors could all contribute to this disparity [36].

In a multivariable logistic regression model, it was revealed that computer skills, EMR training, EMR knowledge, and EMR attitude were all significantly and positively related to EMR readiness. In this study, Health care professionals with computer skills were more likely to be ready to use an EMR system than their counterparts. This finding is supported by previous report in Ethiopia [2, 18, 37], Greek [38], Saudi Arabia [39]. This could be due to the fact that, if used for everyday healthcare management, people with computer capabilities won’t have as much trouble using the EMR system. Additionally, the availability of computers and computer skills may have had a direct impact on health professionals’ perceptions on the usage of computer-based systems. Regarding health professionals who had taken EMR training were about more likely ready for an EMR system as compared to those health professionals who had not taken any EMR training before. This finding was in line with previous studies in Ethiopia [23, 40]. The findings indicating that education and training typically alter people’s perspectives and thoughts may help to explain this.

In this study, health professionals with good knowledge were more likely to be ready for an EMR system than those with poor knowledge. This result was consistent with findings from various studies in Greek [38], Lebanon [41], Australia [42]. This may be explained by the fact that professionals who recognize the benefits of electronic medical record systems could be more encouraged to employ them as a result of their awareness. Because of their tendency to do so, they may also be more prepared to accept technology advantages and be ready to adopt EMR systems.

Moreover, health professionals who had favorable attitude about electronic medical record systems were more likely ready to EMR system than unfavorable attitude towards the system. This may be explained by the fact that experts may be more motivated to use the system if they have a favorable opinion of it and show a keen interest in it. According to earlier research, health professionals’ attitudes influence both their readiness and how well they use the system in Ethiopia [23], Saudi Arabia [39], USA [43]. Furthermore, this could be due to the fact that healthcare providers have a positive opinion of those technologies, which motivates them to be more enthusiastic and dedicated towards using EMR [18, 44].

### Strength and limitation

The strength of this study was the first in its field to analyze the precise measurements that must be made to raise healthcare providers’ readiness levels prior to the deployment of EMR. In low-income nation settings, it also highlighted several key measurements that must be made before ERM implementation. The study was cross-sectional, thus causality cannot be concluded. The study’s primary shortcoming was the lack of triangulation with qualitative results. Additionally, it didn’t include other forms of ready, such as organizational and technological readiness, because there isn’t a tool that can compress all forms of ERM health preparedness.

## Conclusion

Although it was deemed insufficient, more than half of the respondents indicated an acceptable level of overall readiness for the adoption of EMR. According to this study, having computer skills, having EMR training, good EMR knowledge, and favorable EMR attitude were all significantly and positively related to EMR readiness.

Prior to implementation, health personnel should receive training to raise their level of EMR understanding. Capacity building and awareness creation activities should also be made in this regard. Given that it would improve professional abilities and make them feel more competent and ready to use the system, it stands to reason that this could also alter health professionals’ attitudes.

### Implications of the study

Future deployments of digital health systems could be affected by this study. Increasing computer accessibility, providing an EMR training course, and promoting a positive attitude towards EMR are potential strategies to boost the success rate of EMR implementations in Ethiopia. The electronic medical record will be implemented and customized in the study environment using the findings of the study as a foundation. The main goal was to increase the success of EMR implementation by determining the readiness of healthcare professionals.

## Data Availability

Full data set and materials pertaining to this study can be obtained from corresponding author on reasonable request.

## Abbreviations

AOR: Adjusted Odds Ratio
CI: Confidence Interval
EHR: Electronic Health Record
EMR: Electronic Medical Record
ICT: Information Communication Technology
IT: Information Technology
OR: Odds Ratio
